# Hematopoietic Stem Cell Transplantation for Combined Immunodeficiencies, on Behalf of IEWP-EBMT

**DOI:** 10.3389/fped.2019.00552

**Published:** 2020-01-24

**Authors:** Benedicte Neven, Francesca Ferrua

**Affiliations:** ^1^Université de Paris, Paris, France; ^2^Pediatric Hematology-Immunology and Rheumatology Unit, Necker-Enfants Malades Hospital, Assistance Publique—Hôpitaux de Paris (APHP), Paris, France; ^3^INSERM U1163 and Imagine Institute, Paris, France; ^4^San Raffaele Telethon Institute for Gene Therapy (SR-Tiget), Pediatric Immunohematology and Bone Marrow Transplantation Unit, San Raffaele Scientific Institute, Milan, Italy

**Keywords:** HSCT, combined immunodeficiencies, decision to transplant, modalities and timing, CD40L

## Abstract

Combined immunodeficiencies (CIDs) are a clinically and genetically heterogeneous group of primary immunodeficiencies (PIDs) that affect T-lymphocyte immunity with abnormal development or function. As compared to severe combined immune deficiencies (SCID), these patients are usually diagnosed later. They display a broad infectious susceptibility; immune dysregulation manifestations and chronic lymphoproliferation are also frequent. These complications and their specific treatments can lead to persistent damage to several organs. Prognosis of CIDs is worse as compared to other PIDs. The curative treatment is usually hematopoietic stem cell transplantation (HSCT), but difficult questions remain regarding the definitive indication of HSCT and its timing; the final decision depends on a conjunction of factors such as immunological parameters, severity of clinical manifestations, and natural history of the disease, when molecular diagnosis is known. CD40L deficiency, a CID caused by mutations in *CD40LG* gene, well illustrates the dilemma between HSCT *vs*. long-term supportive treatment. This disease leads to higher risk of developing infections from bacterial and intracellular pathogens, especially *Pneumocystis* and *Cryptosporidium spp*. While supportive care allows improved survival during childhood, organ damages may develop with increasing age, mainly chronic lung disease and biliary tract disease (secondary to *Cryptosporidium spp*. infection) that may evolve later to sclerosing cholangitis, a severe complication associated with increased mortality. Early HSCT before organ damage development is associated with best survival and cure rate, while HSCT remains a risky therapeutic option for older patients, for those with organ damage, especially severe liver disease, and/or for those with limited or no donor availability. Prospective studies are needed to analyze risks of HSCT compared to those of life-long supportive therapy, including quality of life measures.

## Heterogeneity of Combined Immunodeficiencies

Combined immunodeficiencies (CIDs) are a clinically and genetically heterogeneous group of primary immunodeficiencies (PIDs) that affect T-lymphocyte immunity with abnormal development or function. Humoral immunity may be intrinsically abnormal or defective as a consequence of an abnormal T-B lymphocyte cooperation (1).

Clinically, CIDs are extremely variable in terms of manifestations, age of onset and severity. A broad susceptibility to opportunistic and non-opportunistic infections including viruses, bacteria, protozoan and parasites is a hallmark of these diseases. Defects of immune regulation are frequent and can lead to autoimmune and immune dysregulation manifestations that in some circumstances can be the predominant features. They occur in up to 40% of patients with CIDs, autoimmune cytopenia and inflammatory bowel diseases being the most frequent (2). Chronic non-malignant lymphoproliferation can occur. It can lead to skin or visceral granulomatous lesions driven in some circumstances by persistent chronic infections such as vaccine-derived rubella virus (3). Increased susceptibility to malignancies, particularly lymphoma is observed (4). This can be due to impaired immunosurveillance to viral or tumoral antigens; chronic inflammation and infection with oncogenic viruses such as Epstein Barr Virus (EBV) can also promote lymphoproliferation. In some PIDs, intrinsic defects of DNA repair may further increase these oncogenic risks. Some CIDs can be associated with syndromic features as microcephaly, dysmorphic features, intrinsic growth retardation (related to osseous dysplasia) (5).

Infectious, autoimmune, inflammatory and oncogenic complications and their specific treatments can lead to persistent damage to several organs including lung, liver, digestive tract, and/or kidney.

There is a continuum between severe combined immune deficiencies (SCID) and CIDs without consensual demarcation between these two groups of diseases (6). CIDs differ by a later age of onset, later age at diagnosis, generally beyond the age of 1 year, and by a higher number of circulating T lymphocytes. Immunological abnormalities are highly variable from profound quantitative or qualitative abnormalities to significant residual T-lymphocyte immunity. Humoral immunity is variably affected. At the other end of the spectrum, common variable immune deficiency (CVID) that occurs later in life is predominantly an antibody deficiency associated in some cases with an impaired T-cell immunity.

Molecular causes of CIDs are numerous and can affect either lymphopoiesis or T-lymphocyte function at several of the numerous developmental, activation or differentiation steps required for an efficient immune response (Table 1) (1). The international Union of Immunological Societies categorized and listed primary immunodeficiencies with known molecular causes (5) and more than 120 gene defects are responsible for CIDs.

**Table 1:** Non excaustive list of CID, their main clinical features and HSCT experiences.

	**Genetic defects**	**Classification in Picard et al. (5)**	**Main clinical features**	**HSCT**	**References**
Defects of T-cell production	Hypomorphic SCID (hypomorphic mutations of genes responsible for typical SCID) - mostly *RAG1, RAG2*.	Table 1	CID with immune-dysregulation, auto-immunity, granuloma	Case reports	(7)
	Hypomorphic mutations in *IL2RG* and *JAK3 genes*	Table 1	CID	Case reports	(8, 9)
	Defects of DNA repair, telomeropathies	Table 2	*See dedicated section*		
Defects of Antigen presentation	Defects of MHC class II expression (*RFXANK, CIITA, RFXAP, RFX5*)	Table 1	*See dedicated section*		
	Defect of MHC class I expression (*TAP1, TAP2, TAPBP, B2M*)	Table 1	Immune-dysregulation, granuloma	Case report	(10)
Defects of proximal TCR signaling	*CD3G*	Table 1	CID with immune-dysregulation, auto-immunity	Case report	(11)
	*TRAC* (*T-cell receptor alpha constant*)	Table 1	CID with immune-dysregulation	Case reports	(12)
	*ZAP70*	Table 1	CID with immune-dysregulation	Case reports	(13)
	*ITK*	Table 1	CID - mainly EBV related diseases	Case reports	(14)
	*LCK*	Table 1	CID with immune-dysregulation, auto-inflammation	Case report	(15)
	*LAT*	Table 1	CID with immune-dysregulation, auto-immunity	Case reports	(16)
	*RHOH*	Table 1	CID - HPV infection	Case reports	(17)
Defects of T-cell activation	Calcium signaling (*STIM1, ORAI1*)	Table 2	CID with immune-dysregulation, auto-immunity	Case reports	(18–20)
	Magnesium signaling (*MAGT1*)	Table 1	CID - EBV related diseases	Case reports	
	Hypomorphic mutation of *IKBKG*/IKK-gamma (NEMO)	Table 2	CID with immune-dysregulation	Small series	(21)
	Gain of function mutation in *NKKBIA (IkBa)*	Table 2	CID with immune-dysregulation	Small series	(22)
	*CARMIL2*	Table 1	CID with immune-dysregulation	Not reported	
	CBM signalosome complex (*CARD11, BCL10, MALT1*)	Table 1	CID with immune-dysregulation	Case reports	(23)
	*MAP3K14* (NIK)	Table 1	CID		(24)
	*IKBKB* (IKK2/IKK-beta)	Table 1	CID		
Defects of T-cell proliferation	*CTPS1*		CID - EBV related diseases	Small series	(25)
Defects of actin cytoskeleton	*WAS* (WASP)	Table 2	*See dedicated section*		(26)
	*WIPF1* (WIP)	Table 2	CID with immune-dysregulation	Case reports	(27)
	*ARPC1B*	Table 2	CID with immune-dysregulation, auto-immunity	Case reports	(28)
	*DOCK8*	Table 1	*See dedicated section*		(26)
	*DOCK2*	Table 1	CID with immune-dysregulation	Case reports	(29)
	*CORO1A* (Coronin 1A)	Table 1	CID - EBV related diseases	Case reports	(30, 31)
	*MSN* (Moesin)	Table 1	CID, auto-immunity	Case reports	(32)
	*RAC2 (dominant negative mutation)*	Table 1	Lymphopenia, defective neutrophil chemotaxis	Case reports	(33)
	*RAC2 (homozygous loss of function mutation)*	Table 1	CVID, autoimmunity	No HSCT reported	(34)
	*RAC2 (gain of function mutations)*	Table 1	CID	Case reports	
Defects of co-stimulatory molecules	*CD27*	Table 4	CID with immune dysregulation and EBV related diseases	Case reports	
	*CD70*	Table 4	CID with immune dysregulation and EBV related disease	Case reports	
	*TNFRSF9* (4-1BB, CD137)		CID with immune dysregulation and EBV related disease	Case reports	
	*TNFRSF4* (OX40)	Table 1	CID with HHV8-Kaposi sarcoma	No HSCT reported	
	*IL21-1L21R*	Table 1			
	*CD40*	Table 1	CID	Small series	(35)
	*CD40LG*	Table 1	CID		
Defects of regulatory function and other diseases of immune dysregulation	IPEX (*FOXP3*), *IL2RA (CD25), IL10, IL10R1, IL10R2, CTLA4, LRBA*	Table 4	*See dedicated section*		
	*PIK3CD* (GOF), *PIK3R1* (LOF)	Table 3	CID with immune-dysregulation, auto-immunity	Small series	(36)

*ID, immunodeficiencies, CID, combined immunodeficiencies, SCID, severe combined immunodeficiencies. Table 1: ID affecting cellular and humoral immunity; Table 2: CID with associated or syndromic features; Table 3: Predominantly antibody deficiencies; Table 4: Diseases of immune dysregulation (susceptibility to EBV and lymphoproliferative conditions)*.

Thanks to improved performance of next generation sequencing, there has been a rapid expansion in the identification of causative gene alterations among patients with CIDs in recent years. Genetic diagnostic is also shortened by these new approaches. However, a proportion of patients with CIDs remains without molecular diagnosis (37).

## Hematopoietic Stem Cell Transplantation in CIDs

### Indication to Transplant

Prognosis of CIDs is worse as compared to other PIDs such as humoral defects, innate, and granulocyte immune deficiencies (38). The curative treatment is often hematopoietic stem cell transplantation (HSCT) that has been increasingly successful in recent years. However, the broad spectrum of genetic defects underlying CIDs, the large range of T-cell defects and clinical heterogeneity in each disease make the decision about transplantation very challenging and prevent from universal recommendations regarding indication, timing and modalities of HSCT. Rather, it invites to disease and patient tailored treatment (39) that integrates and weights all these parameters including the characteristics of available donors to balance the final decision.

For patients with early severe clinical presentations mimicking SCID, the recommendation to drive the patient quickly to HSCT will be straightforward, including with alternative donors. However, no threshold of T-cell immunity required to prevent severe complications has been established. When molecular diagnosis is known, it may guide the decision for diseases with well-known natural history, helping to weight the balance between risks of disease evolution with potential development of future organ damage vs. risks of transplantation. However, many genetic causes of CIDs have been recently described, involving a small number of individuals with limited experiences, as summarized in Table 1. In these cases, natural history studies are needed. Several retrospective studies co-ordinated by the Inborn Error Working Party (IEWP) of the European Society for Bone and Marrow Transplantation (EBMT) are ongoing (e.g., in hypomorphic RAG1 and RAG2 deficiencies, CD27/CD70 deficiencies) and will help to define the best treatment strategies in these conditions. When the molecular diagnosis is unknown, the decision to proceed to transplant is mostly based on clinical judgement.

Retrospective studies performed in specific CIDs [Wiskott-Aldrich syndrome (WAS), MHCII expression deficiency, CD40L deficiency, dedicator of cytokinesis 8 (DOCK8) deficiency] (40–42) advocate for early transplantation before organ damage development or occurrence of severe, chronic infections [see reviews on Hematopoietic stem cell transplantation for WAS and DOCK8 deficiency (26) and for MHC class II expression deficiency]. Similar recommendations may apply more broadly to CIDs. The p-CID study, a prospective international multicentre observational study that is currently recruiting is designed to try to define clinical events and immunologic parameters predictive of outcome with the aim to help making the decision to transplant in these conditions (39).

The donor availability needs also to be considered for the transplant decision. Whatever the diagnosis, HSCT from a healthy matched sibling donor (MSD) remains the gold standard that offers the best results and allows to consider HSCT early in the course of the disease, even pre-emptively if poor outcome is predictable (38) [see reviews on Hematopoietic stem cell transplantation for WAS and DOCK8 deficiency (26) and for MHC class II expression deficiency]. Outcomes obtained with matched unrelated donors (MUD) have increasingly improved in the last decade and are approaching similar survival rates as with MSD [albeit with higher rate of HSCT-related complications (41)] [see review on Hematopoietic stem cell transplantation for WAS and DOCK8 deficiency (26)], allowing extended indications. In the absence of a matched-donor, decision to transplant needs to be balanced. Transplantation from an alternative donor is more challenging even though most recent results show increasingly successful survival, allowing to consider transplant early in the disease course (43–46). At the end, the final decision about transplant will depend on a conjunction of factors such as immunological parameters, the severity of past and current clinical manifestations, i.e., severity of infections, autoimmunity, need of immunosuppressive treatment to control the manifestations related to immune dysregulation and donor compatibility. Family history and their experience with the disease will also weight in the final decision.

### Pre-HSCT Evaluation

Cautious evaluation by a multi-disciplinary team is important before transplantation. Previous recurrent or chronic infections may have led to severe and irreversible visceral damage, notably lung and liver. Renal impairment can also occur as a consequence of chronic or recurrent anti-infectious treatment or prolonged immunosuppression. Aggressive pre-emptive treatment and control of all treatable pre-existing infections needs to be proposed prior HSCT. Special attention to detect emergence of antimicrobial drug resistance need to be given, especially in heavily pre-treated patients. In case of EBV-related lymphoproliferation prior to HSCT, the degree of remission required before transplant is a question of debate (47). Optimization of nutritional status is also important. Remission of pre-existing autoimmunity and/or inflammatory conditions should as much as possible be obtained before transplantation, while avoiding prolonged heavy immunosuppression both before and after HSCT that may increase infectious susceptibility.

### Conditioning Regimen and GVHD Prophylaxis

The residual T-cell immunity and the intact hematopoiesis of these patients increases the risk of allograft rejection, particularly in case of HLA disparity between donor and recipient. Some degree of myeloablation is usually required. Development of reduced toxicity conditioning regimens (CR) has contributed to reduce transplant-related mortality (TRM) in the last decade (48, 49). Currently, the most frequent associations proposed from the more myeloablative to the more reduced intensity are busulfan/fludarabine, treosulfan/fludarabine, and fludarabine/melphalan. In the absence of organ damage, myeloablative conditioning (MAC) is preferred, while reduced intensity conditioning (RIC) will be considered in case of pre-HSCT co-morbidities. Addition of tiothepa to treosulfan/fludarabine can be discussed in case of mismatched transplant, especially if an *ex vivo* manipulation of the graft is proposed. Addition of serotherapy (anti-thymocytes globulins or alemtuzumab) is frequently recommended to prevent rejection and GVHD, but its omission can be discussed in HSCT from a MSD. In case of pre-HSCT autoimmunity, treatment with anti-CD20 monoclonal antibodies can be proposed.

Full donor chimerism in all lineages is not always mandatory to correct the underlying disease with exceptions such as WAS, where mixed chimerism has been related to inferior long-term outcome (autoimmunity and thrombocytopenia) (45, 50).

The risk of occurrence of graft-vs.-host-disease (GVHD) is dependent on the degree of compatibility between donor and recipient, but also on the clinical conditions of the recipient. Pre-existing viral infections or inflammation related to autoimmunity or immune dysregulation can predispose to onset of early and severe acute GVHD and therefore, effective GVHD prophylaxis is of primary importance. If GVHD occurs, it needs to be aggressively and rapidly treated.

### Donor Choice

HSCT from a geno-identical healthy sibling remains the best option, but available in fewer than 25% of cases. A suitable MUD will be available in ~70% of the remaining patients, or even less for patients belonging to ethnic groups insufficiently represented in donor registries (51). Options will be HSCT from an alternative donor, i.e., unrelated mismatched cord-blood (MMCB), mismatched unrelated donor (MMUD), or HLA-haploidentical mismatched family donor (MMFD). In MMUD and MMFD, different types of graft manipulations have been developed to reduce risks of GVHD and graft rejection (52). Historically, CD34+ cell selection was the most commonly used technique, but non-engraftment and slow immune reconstitution limited its applications especially in CIDs. New strategies with selective depletions as CD3/CD19 or TCRalpha/beta and CD19 depletions are increasingly used, limiting the risk of GVHD and allowing good engraftment rate, as long as a myeloablative conditioning (MAC) is administered (53, 54). However, poor early immune reconstitution might still be problematic notably in patients with active infection at the time of HSCT. Haplo-HSCT strategies without *ex vivo* graft manipulation, but using post-transplant cyclophosphamide (PTCY) as GVHD prophylaxis, have been increasingly used to treat adult patients with malignant diseases. Data on PTCY in children with PIDs and CIDs are still limited but encouraging (43, 44). All these alternative approaches need to be prospectively compared in the context of CIDs.

In conclusion, early molecular diagnosis to facilitate early decision of transplantation before development of co-morbidities, tailoring conditioning regimen to reduce toxicity, improving GVHD prophylaxis and kinetics of immune reconstitution while improving supportive care, are future perspectives that should contribute to improvement of HSCT results in CIDs.

## The Example of CD40L Deficiency: HSCT vs. Long-term Supportive Treatment

CD40 ligand (CD40L) deficiency (55, 56) is a X-linked disease related to hemizygous mutations in *CD40LG* gene, encoding CD40L, a glycoprotein (5, 57–61) mainly involved in co-stimulatory T-lymphocyte function. Impaired CD40L expression alters B-cell isotype switching and antibody production, dendritic cell signaling, and myeloid cell function and development (62, 63). Generally, disease onset occurs during early childhood, with frequent respiratory tract infections, diarrhea and neutropenia. As a consequence of chronic cryptosporidial infection, severe biliary tract disease may develop and evolve to sclerosing cholangitis.

CD40L deficiency is associated with high morbidity and mortality. Historically, long-term survival has been poor with supportive therapy only, with 20–50% of patients reaching their thirties (64–66) and only sporadic observations of long-term survivors (67, 68). A median survival of 25 years from diagnosis has been recently shown in 109 patients treated with conservative therapy only (67). This approach currently includes immunoglobulin replacement (IgR), antimicrobial prophylaxis, close monitoring for complications, and careful precautions to avoid Cryptosporidium species exposure. Liver disease is the most significant predictor of mortality (67, 69) and, together with severe infections, represents the major cause of death (64).

At present, HSCT represents the only curative treatment for this disease. Historically, its outcome has not been optimal (65). In a first European retrospective survey (70), overall survival (OS) after HSCT was 68% and transplant was curative in only 58% of patients, most of whom without liver disease. More recently, the multi-center retrospective observational study of de la Morena et al. showed similar OS between patients with CD40L deficiency treated with or without HSCT, but among survivors, transplanted patients showed higher median age performance status scores and greater well-being, as compared to non-transplanted patients (67). In addition, a survival improvement for transplanted patients was progressively observed since 1987, suggesting better transplant practices in more recent years (67). This positive trend was confirmed by a recent Japanese study (71), reporting improved outcome in 29 patients undergoing HSCT, and also by a subsequent international retrospective survey (42), in which HSCT outcome was analyzed in 130 patients with CD40L deficiency transplanted between 1993 and 2015. In this study, OS, event-free (EFS) and disease-free survival (DFS) 5 years post-HSCT were 78.2, 58.1, and 72.3%, respectively. A general HSCT outcome improvement was confirmed after 2000, mainly thanks to amelioration of transplant-related procedures, earlier age at transplantation, and lower organ damage burden, especially liver disease. Indeed, after 2000, best results were obtained in children transplanted before 5 years, while organ damage before HSCT was confirmed to negatively influence outcome (42). Superior OS was achieved with matched donors, both MSD and MUD. Myeloablation resulted in better OS and DFS, as compared to RIC. Moreover, MAC was associated with higher EFS. Among survivors who could stop IgR, T-lymphocyte chimerism was mainly donor in most. However, decreasing lineage-specific chimerism was observed in some transplants, underlying the problem of residual host immunity. In line with this, myeloablation seemed to be required to attain complete engraftment. Indeed, RIC and non-myeloablative conditioning resulted associated with poor or absent engraftment.

Interestingly, de la Morena et al. (67) evidenced geographic differences in HSCT practices: indication for HSCT was variable, with higher transplant rates in European centers as compared to American ones. This is a reflection of the still unsolved debate about the best management of these patients. Guidelines were proposed by the European Society for Blood and Marrow Transplantation/European Society for Immunodeficiencies Inborn Errors Working Party in 2011 (72). These recommendations favored HSCT at diagnosis in case of MSD availability, HSCT at early complication development in case of MUD or MMUD, and later, salvage HSCT in case of progressive organ damage for those with MMFD available only. However, recently published data prompt an update of these guidelines, suggesting that HSCT can cure CD40L deficiency, but its outcome is better if it is performed upfront, before the development of organ damage, and using MAC, which associated with higher survival and cure rate. In addition, it was recently shown (73) that an effective early HSCT can also improve or even reverse mild to moderate chronic cholangiopathy, presumably thanks to the clearance of chronic infections after immune reconstitution. Nevertheless, in spite of recent outcome amelioration, HSCT still remains a risky therapeutic option for older patients, for those with organ damage, especially severe liver disease, and/or for those with limited donor availability. For these patients, gene editing may represent an attractive potential alternative treatment, since infusion of gene-corrected T-lymphocytes or HSC may be curative (74, 75) [see review on “Autologous stem cell-based gene therapy for inherited disorders: state-of-the-art and future prospects” (76)]. Finally, prospective studies are needed to perform comparative analyses about the risks of HSCT and those of life-long supportive therapy, including quality of life measures. Detailed longitudinal clinical data are lacking, especially regarding infection-related burden in terms of frequency of hospital admissions or missed days of school/work. These are fundamental to standardize patients' management aimed not only at improving survival, but also at optimizing quality of life.

## Author Contributions

All authors listed have made a substantial, direct and intellectual contribution to the work, and approved it for publication.

### Conflict of Interest

The authors declare that the research was conducted in the absence of any commercial or financial relationships that could be construed as a potential conflict of interest.
